# ZDHHC11 Positively Regulates NF-κB Activation by Enhancing TRAF6 Oligomerization

**DOI:** 10.3389/fcell.2021.710967

**Published:** 2021-08-19

**Authors:** Enping Liu, Jiawei Sun, Jing Yang, Lin Li, Qili Yang, Jiuqin Zeng, Jiayu Zhang, Dahua Chen, Qinmiao Sun

**Affiliations:** ^1^State Key Laboratory of Membrane Biology, Institute of Zoology, Chinese Academy of Sciences, Beijing, China; ^2^Institute of Stem Cells and Regeneration, Chinese Academy of Sciences, Beijing, China; ^3^School of Life Sciences, University of Chinese Academy of Sciences, Beijing, China; ^4^Institute of Physical Science and Information Technology, Anhui University, Hefei, China; ^5^Institute of Biomedical Research, Yunnan University, Kunming, China

**Keywords:** ZDHHC11, NF-κB, TRAF6, oligomerization, inflammation

## Abstract

Tumor necrosis factor receptor-associated factor 6 (TRAF6) is a RING domain ubiquitin ligase that plays an important role in nuclear factor-κB (NF-κB) signaling by regulating activation of the TAK1 and IKK complexes. However, the molecular mechanisms that regulate TRAF6 E3 activity remain unclear. Here, we found that ZDHHC11, a member of the DHHC palmitoyl transferase family, functions as a positive modulator in NF-κB signaling. ZDHHC11 overexpression activated NF-κB, whereas ZDHHC11 deficiency impaired NF-κB activity stimulated by IL-1β, LPS, and DNA virus infection. Furthermore, Zdhhc11 knockout mice had a lower level of serum IL6 upon treatment with LPS and D-galactosamine or HSV-1 infection than control mice. Mechanistically, ZDHHC11 interacted with TRAF6 and then enhanced TRAF6 oligomerization, which increased E3 activity of TRAF6 for synthesis of K63-linked ubiquitination chains. Collectively, our study indicates that ZDHHC11 positively regulates NF-κB signaling by promoting TRAF6 oligomerization and ligase activity, subsequently activating TAK1 and IKK complexes.

## Introduction

The nuclear factor-κB (NF-κB) transcription factor not only plays important roles in mediating immune responses, cell proliferation and death, but also is critical for inflammatory responses (Taniguchi and Karin, 2018). It can be activated by stimulation of various receptors such as Toll-like receptors (TLRs), IL-1 receptor (IL-1R), TNFR1, RIG-I-like receptors, and cGAS (Yoneyama et al., 2004; Verstrepen et al., 2008; Hopfner and Hornung, 2020). IL-1β/IL-1R and LPS/TLR4-mediated NF-κB signaling share a similar signaling pathway. Receptor IL-1R as well as TLR4 recruit the adaptor protein MyD88 after binding to ligand IL-1 or LPS, respectively, then MyD88 forms complex with IL-1R-associated kinases (IRAKs), including IRAK1, IRAK2, and IRAK4, and Tumor necrosis factor receptor-associated factor 6 (TRAF6; Janssens and Beyaert, 2002). Once the MyD88 complex is activated, TRAF6 servers as an E3 ubiquitin ligase to catalyze the synthesis of K63-linked polyubiquitin chains conjugated to itself or other proteins or as free ubiquitin chains (Shi and Sun, 2018). K63-linked polyubiquitin chains bind to TAB2 and NEMO to recruit TAK1-TAB1-TAB2 or TAB3 and the IκB kinase IKKα-IKKβ-NEMO complex, respectively, which facilitates TAK1 and IKK activation (Kanayama et al., 2004). Activated TAK1 triggers IKK complex activation and then the IKK complex phosphorylates IκB protein that binds to NF-κB in the cytoplasm of resting cells, which results in its ubiquitination and degradation. NF-κB is released from association with IκB, and then translocates to the nucleus to trigger transcription of proinflammatory cytokines such as tumor necrosis factor (TNFα), IL-1β, IL-8, and IL-6 (Adhikari et al., 2007; Mulero et al., 2019).

Tumor necrosis factor receptor-associated factor 6 belongs to the tumor necrosis factor receptor-associated factor (TRAF) family, and plays important roles in activation of NF-κB signaling by IL-1β and LPS (Cao et al., 1996; Lomaga et al., 1999). TRAF6 is also involved in regulating the activation of NF-κB signaling induced by virus infection. Previous studies showed that while TRAF6 plays a redundant role with TRAF2 and TRAF5 in activating NF-κB induced by RNA virus infection (Liu et al., 2013), TRAF6 is critical for the activation of NF-κB induced by DNA virus infection (Abe and Barber, 2014). In NF-κB signaling, TRAF6 acts as an E3 ubiquitin ligase together with the Ub-conjugating enzyme (E2) complex Ubc13-Uev1A to synthesize polyubiquitin chains linked through Lys-63 (K63) of Ub (Deng et al., 2000). The polyubiquitin chains of K63-linked ubiquitin bind to TAB2 and NEMO, which activates the TAK1 and IKK complex, respectively, Deng et al. (2000), Kanayama et al. (2004), and Wu et al. (2006). TRAF6 harbors an N-terminal RING finger domain, followed by Zn Finger domains and the C-terminal TRAF domain (Xie, 2013). The N-terminal RING and ZF1 domains constitute the minimal unit to catalyze K63-linked polyubiquitin chain synthesis *in vitro*, and the C-terminal TRAF-C domain facilitates its oligomerization and association with receptors and adaptor proteins (Ye et al., 2002; Fu et al., 2018). TRAF6 oligomerization is important for its E3 activity (Yin et al., 2009; Fu et al., 2018). A previous study has shown that TIFA promotes TRAF6 oligomerization and ubiquitination (Ea et al., 2004). Although previous studies have made significant progress in delineating the functions of TRAF6, the mechanism of TRAF6 regulation remains unclear.

Protein S-palmitoylation is a reversible post-translational modification that is dynamically controlled by palmitoyl acyl transferases and palmitoyl thioesterase (Yount et al., 2013; Zaballa and van der Goot, 2018). Palmitoylated proteins are involved in regulating numerous protein properties including trafficking, localization, stability, activity, and association with other proteins (Daniotti et al., 2017; Ko and Dixon, 2018). The majority of protein palmitoylation is catalyzed by the family of palmitoyl acyl transferases that have a zinc finger DHHC (ZDHHC) domain required for palmitoyl transfer activity (Mitchell et al., 2006). There are 23 members of the diverse DHHC protein family in humans, which include ZDHHC1-ZDHHC24 (ZDHHC10 is omitted) in humans (Korycka et al., 2012; Lemonidis et al., 2015). Previous studies have shown that ZDHHC11 is an ER-associated protein and that its aberrant expression is related to the development of some cancers (Yamamoto et al., 2007; Kang et al., 2008; Gorleku et al., 2011; Wu et al., 2013; Dzikiewicz-Krawczyk et al., 2017). Recently, ZDHHC11 was found to regulate innate immune responses against DNA virus infection by mediating the MITA–IRF3 association (Liu et al., 2018). However, whether ZDHHC11 is involved in inflammatory pathways remains unclear.

In the present study, we identified ZDHHC11 as a positive modulator in NF-κB signaling, and found that ZDHHC11 was involved in regulating the activity of NF-κB stimulated by IL-1β, LPS, and DNA virus infection. *Zdhhc11*^–/–^ mice exhibited a lower level of serum IL-6 upon treatment with LPS and D-galactosamine or HSV-1 infection than control mice. Moreover, we demonstrated that ZDHHC11 associated with TRAF6 and then increased TRAF6 oligomerization, which enhanced TRAF6 E3 activity to synthesize K63-linked ubiquitination chains. Taken together, we found that ZDHHC11 increases oligomerization and E3 activity of TRAF6, which leads to activation of TAK1 and IKK, and then positively modulates NF-κB signaling.

## Results

### ZDHHC11 Positively Regulates IL-1β-Induced NF-κB Activation

**FIGURE 1:**
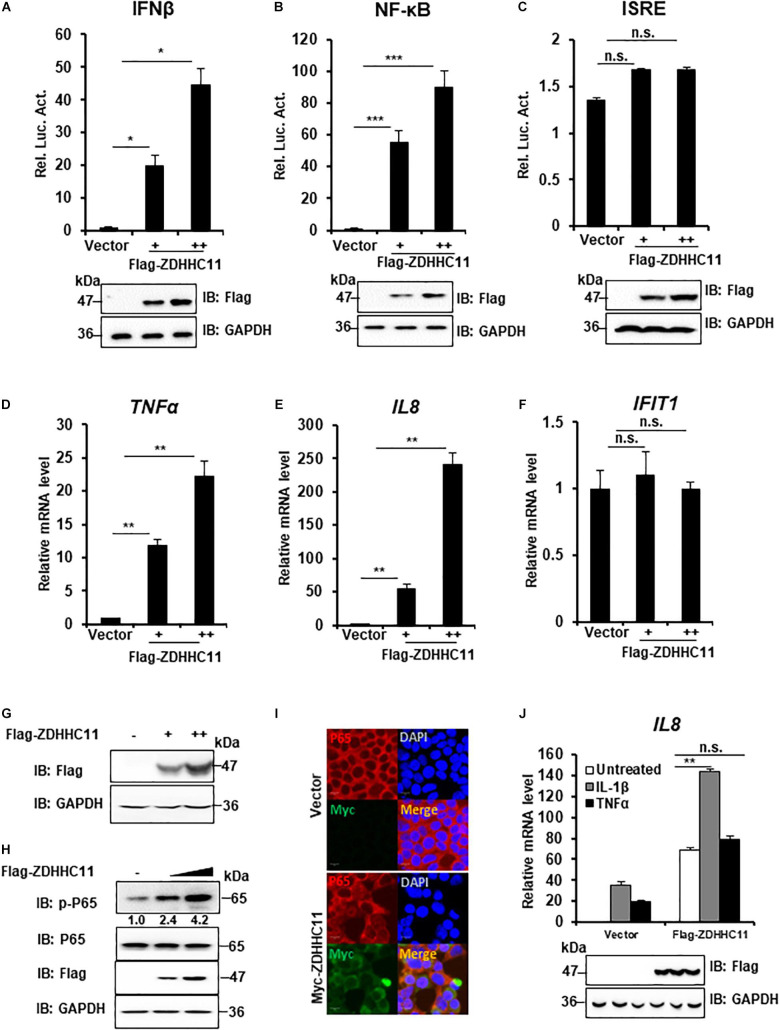
ZDHHC11 overexpression activates NF-κB signaling. **(A–C)** HEK293T cells were cotransfected with an empty vector or different doses of ZDHHC11 (50 and 100 ng) expression plasmids and luciferase reporter plasmids IFN-β-Luc (100 ng; **A)**, NF-κB-Luc (20 ng; **B)**, or ISRE-Luc (100 ng; **C)**. After 24 h, the cells were harvested for luciferase reporter assay (upper panel) and immunoblot assay (lower panels). **(D–G)** HEK293T cells were transfected with empty vector or different amount of ZDHHC11 expression plasmids (50 and 100 ng). After 24 h, the cells were harvested to isolate RNA or protein, followed by qRT-PCR analysis to measure the mRNA levels of *TNF*α **(D)**, *IL8* **(E)**, and *IFIT1* **(F)** or immunoblotting to detect ZDHHC11 protein expression **(G)**, respectively. **(H)** HEK293T cells were transfected with different doses of ZDHHC11 expression plasmids (50 and 100 ng) or empty vector. After 24 h, the cells were harvested for immunoblot assay with the corresponding antibodies. **(I)** HEK293T cells were transfected with Myc-tagged ZDHHC11 expression plasmids (200 ng) or empty vector. After 24 h, the cells were fixed, stained with the indicated antibodies. The images were taken by confocal microscopy. Scale bars, 10 μm. **(J)** HEK293 C6 cells were transfected with indicated plasmids (200 ng). After 24 h, the cells were untreated or treated by IL-1β (10 ng/ml) or TNFα (10 ng/ml) for 10 h, followed by qRT-PCR analysis (upper panel) or immunoblot assay (lower panels). Data shown in **(A–F,J)** are representative of three independent experiments (mean ± SD of duplicate experiments). *P < 0.05; **P < 0.01; ***P < 0.001; n.s. not significant versus the control groups; and Student’s *t*-test. All blots are representative of three independent experiments.

To determine whether ZDHHC family members play a role in innate immune signaling, HEK293T cells were cotransfected 16 independent cDNA expression plasmids that encoded members of the ZDHHC family with a luciferase gene under the control of the IFNβ promoter (IFNβ-Luc), which contains NF-κB and IRF3 binding sites. As a result, we found that ZDHHC11 significantly induced IFNβ activity compared with other ZDHHC family members (Supplementary Figure 1). Because IFN-β induction requires the coordinated action of both IRF3 and NF-κB, we next investigated how ZDHHC11 activated the IFNβ promoter. We employed an NF-κB luciferase reporter and IFN-stimulated response element (ISRE) luciferase reporter that is activated by IRF3. Reporter assays showed that ZDHHC11 overexpression activated IFNβ and NF-κB, but not the ISRE reporter, in a dose-dependent manner in HEK293T cells (Figures 1A–C). Consistently, quantitative reverse transcription-PCR (qRT-PCR) assays demonstrated that ZDHHC11 overexpression in HEK293T cells increased the mRNA levels of NF-κB downstream genes *TNF*α and *IL8*, but not IRF3-dependent genes, such as *IFIT1* (Figures 1D–G). Similarly, ZDHHC11 overexpression increased the mRNA levels of *TNF*α, *IL6*, and *IL8* in HeLa cells (Supplementary Figures 2A–D). Because activation of NF-κB signaling induces p65 phosphorylation and nuclear translocation, we next examined whether ZDHHC11 overexpression affected these characteristics. Western blotting results showed that ZDHHC11 overexpression increased the level of phosphorylated p65 in a dose-dependent manner (Figure 1H and Supplementary Figure 2E) and p65 nuclear translocation was induced when ZDHHC11 was overexpressed in HEK293T cells (Figure 1I). These data suggest that ZDHHC11 overexpression specifically activates NF-κB signaling.

Proinflammatory cytokine interleukin-1β (IL-1β) and TNFα trigger NF-κB activation, next, we tried to determinate whether ZDHHC11 played a role in regulating IL-1β- and TNFα-mediated NF-κB activation. qRT-PCR assays indicated that ZDHHC11 overexpression significantly increased the *IL8* mRNA level induced by IL-1β, but not TNFα, in HEK293 C6 cells that ectopically express IL-1R (Figure 1J). These data suggest that ZDHHC11 specifically enhances NF-κB activation triggered by IL-1β.

### ZDHHC11 Knockdown Decreases IL-1β-Induced NF-κB Activation

**FIGURE 2:**
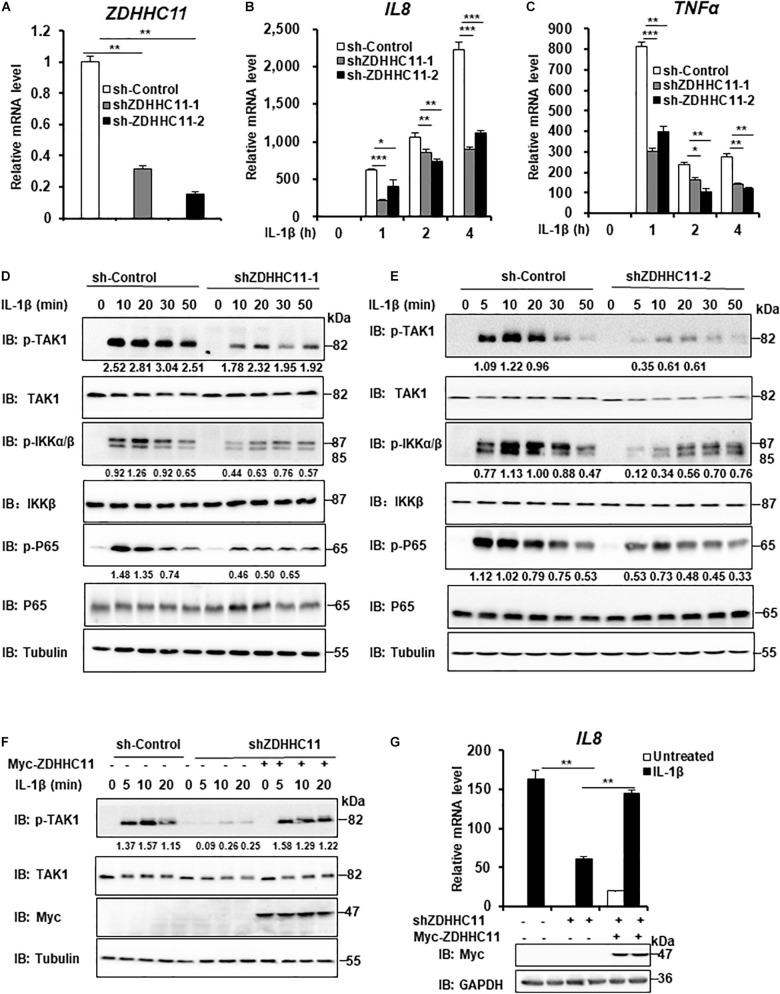
ZDHHC11 knockdown attenuates IL-1β-induced NF-κB activation. **(A–C)** HEK293 C6 cells were infected with lentivirus expressing shRNA that targeted different coding regions of human *ZDHHC11* (shZDHHC11-1 and shZDHHC11-2) or an empty vector for 48 h and then unstimulated or stimulated with IL-1β (10 ng/ml) for the indicated times. qRT-PCR assays were performed to examine the mRNA levels of *ZDHHC11* **(A)**, *IL8* **(B)**, and *TNF*α **(C)**. **(D,E)** Infection was performed as described in **(A)**. The cells were unstimulated or stimulated with IL-1β (10 ng/ml) for the time as indicated. Cells were harvested to perform immunoblot assay. **(F)** HEK293 C6 cells were infected with lentivirus expressing shRNA that targeted *ZDHHC11* or an empty vector for 48 h and then transfected with a ZDHHC11 expression plasmid (500 ng) or empty vector as indicated. After 24 h of transfection, the cells were simulated with IL-1β (10 ng/ml) for the indicated times, followed by immunoblot analysis. **(G)** HEK293 C6 cells were infected and transfected as described in **(F)** and then treated with IL-1β for 3 h, followed by qRT-PCR analysis. Data shown in **(A–C,G)** are representative of three independent experiments (mean ± SD of duplicate experiments). *P < 0.05; **P < 0.01; ***P < 0.001 versus the control groups; and Student’s *t*-test. All blots are representative of three independent experiments.

To further determinate the biological functions of endogenous ZDHHC11 in modulating NF-κB activation, we employed two lentivirus-delivered shRNAs that specifically targeted non-overlapping regions of the coding region of human *ZDHHC11* and evaluated whether knockdown of *ZDHHC11* affected NF-κB signaling in HEK293 C6 cells. As shown in Figures 2A–C, both shRNA-ZDHHC11-1/2 efficiently reduced the level of *ZDHHC11* mRNA and knockdown of *ZDHHC11* significantly reduced transcriptional levels of *IL8* and *TNF*α after IL-1β stimulation in a time-independent manner compared with control cells. Consistently, the levels of phosphorylated TAK1, IKKα/β, and p65 were decreased in *ZDHHC11* knockdown cells after IL-1β stimulation (Figures 2D,E and Supplementary Figure 3A). To determine the specific role of ZDHHC11, we conducted rescue experiments, and observed that restored expression of ZDHHC11 reversed the reduced levels of phosphorylated TAK1 and *IL8* mRNA induced by IL-1β stimulation in ZDHHC11 knockdown cells (Figures 2F,G). Additionally, we performed a similar knockdown assay in HeLa cells and obtained similar qRT-PCR results as those in HEK293 C6 cells (Supplementary Figures 3B–D). Western blotting also indicated that knockdown of *ZDHHC11* reduced the levels of phosphorylated TAK1, IKKα/β, and IκBα stimulated by IL-1β (Supplementary Figures 3E,F). Taken together, these results support the notion that ZDHHC11 positively modulates the NF-κB signaling.

### *Zdhhc11* Deficiency Reduces NF-κB Activation Stimulated by IL-1β and LPS Treatments as Well as DNA Virus Infection

**FIGURE 3:**
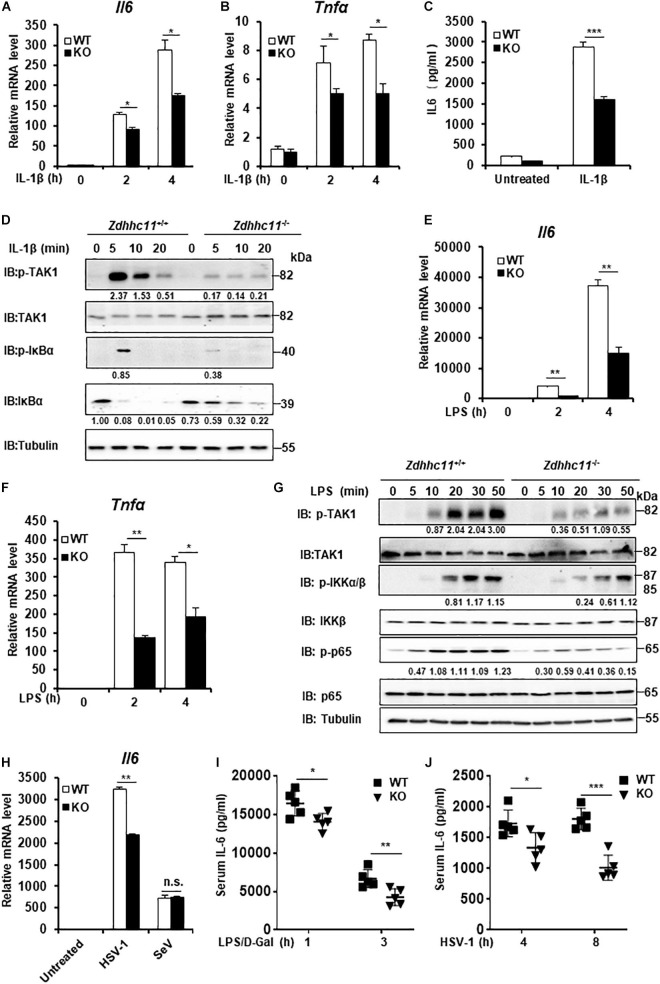
*Zdhhc11* deficiency reduces activation of NF-κB stimulated by multiple stimuli. **(A,B)** *Zdhhc11*^+/+^ and *Zdhhc11*^–/–^ MEFs were stimulated with IL-1β (20 ng/ml) for the time as indicated, then qRT-PCR assay was performed to measure transcriptional levels of *Il6* **(A)** and *Tnf*α **(B)**. **(C)** *Zdhhc11*^+/+^ and *Zdhhc11*^–/–^ MEFs were stimulated with IL-1β (2 ng/ml) for 18 h, followed by an ELISA Kit to measure IL6 in the culture supernatant. **(D)** *Zdhhc11*^+/+^ and *Zdhhc11*^–/–^ MEFs were treated with IL-1β (20 ng/ml) for the indicated times, followed by immunoblot analysis. **(E,F)** *Zdhhc11*^+/+^ and *Zdhhc11*^–/–^ BMDMs were treated with LPS (20 ng/ml) for 0, 2, or 4 h and then analyzed by qRT-PCR assays to examine transcriptional levels of *Il6* **(E)** and *Tnf*α **(F)**. **(G)** *Zdhhc11*^+/+^ and *Zdhhc11*^–/–^ BMDMs were treated with LPS (30 ng/ml) for the indicated times. The cell extracts were harvested for immunoblot analysis. **(H)** *Zdhhc11*^+/+^ and *Zdhhc11*^–/–^ BMDMs were infected with HSV-1 (5MOI) or SeV (10HA) for 6 h, then qRT-PCR assays were performed to measure the mRNA level of *Il6*. **(I)** *Zdhhc11*^+/+^ and *Zdhhc11*^–/–^ mice (*n* = 5) were treated with LPS and D-galactosamine via intraperitoneal injection. Sera were collected at 1 and 3 h after injection to measure IL-6 levels by an ELISA Kit. **(J)** *Zdhhc11*^+/+^ and *Zdhhc11*^–/–^ mice (*n* = 5) were infected with HSV-1 via intravenous injection at 2 × 10^7^ PFU per mouse. Sera were collected at 4 and 8 h after infection to measure IL-6 by an ELISA kit. Data shown in **(A–C,E,F,H)** are representative of three independent experiments (mean ± SD of duplicate experiments). *P < 0.05; **P < 0.01; ***P < 0.001; n.s. not significant versus the control groups; and Student’s *t*-test. All blots are representative of three independent experiments.

To further elucidate the physiological roles of ZDHHC11 in NF-κB activation, we employed *Zdhhc11*-deficient mice from Jackson Lab. We generated mouse embryonic fibroblasts (MEFs) from *Zdhhc11*^+/+^ and *Zdhhc11*^–/–^ 13.5-day-old embryos by breeding heterozygote mutants (Supplementary Figure 4A) and then examined the effect of *Zdhhc11* deficiency on NF-κB signaling. qRT-PCR showed that *Zdhhc11* knockout significantly reduced the mRNA levels of *Il6* and *Tnf*α after stimulation by IL-1β compared with WT controls (Figures 3A,B). Consistently, an enzyme linked immunosorbent assay (ELISA) showed that IL6 protein induced by IL-1β was lower in *Zdhhc11*^–/–^ MEFs than in *Zdhhc11*^+/+^ control cells (Figure 3C). Additionally, western blotting indicated that *Zdhhc11* knockout in MEFs reduced the levels of phosphorylated TAK1, IκBα, and IκBα degradation after IL-1β stimulation (Figure 3D). Next, we determined the effect of *Zdhhc11* deficiency on NF-κB signaling in bone marrow-derived macrophages (BMDMs). qRT-PCR assays demonstrated that the mRNA levels of *Il6* and *Il-1*β were significantly attenuated in *Zdhhc11*^–/–^ BMDMs compared with *Zdhhc11*^+/+^ cells after IL-1β stimulation (Supplementary Figures 4B,C). These findings indicate that ZDHHC11 plays a critical role in IL-1β-triggered NF-κB activation in MEFs and macrophages.

Considering that both LPS and IL-1β trigger NF-κB activation and share similar intracellular signaling pathways (Narayanan and Park, 2015), we next determined whether ZDHHC11 is involved in LPS-induced NF-κB activation. qRT-PCR assays indicated that the mRNA levels of *Il6* and *Tnf*α after LPS stimulation were significantly lower in *Zdhhc11*^–/–^ MEFs (Supplementary Figures 4D,E) and BMDMs (Figures 3E,F) compared with their *Zdhhc11*^+/+^ counterparts. ELISA results also showed that Zdhhc11 deficiency reduced the production of IL6 and TNFα induced by LPS stimulation in BMDMs (Supplementary Figures 4F,G). Consistently, the levels of phosphorylated TAK1, IKKα/β, and p65 in *Zdhhc11*^–/–^ BMDMs were lower than those in *Zdhhc11*^+/+^ BMDMs (Figure 3G). These findings indicate that ZDHHC11 also plays a critical role in LPS-triggered NF-κB activation.

Next, we examined whether ZDHHC11 was involved in NF-κB activation induced by virus infection. As shown in Figure 3H, *Zdhhc11* deficiency in BMDMs decreased the mRNA level of *Il6* induced by infection with herpes simplex virus (HSV-1), but it had no effect on the expression of *Il6* mRNA stimulated by Sendai virus, a kind of RNA virus. Consistent with the results in BMDMs, mRNA levels of *Il6* and *Tnf*α were also lower in Zdhhc11^–/–^ MEFs than wild-type cells upon HSV-1 infection (Supplementary Figures 4H,I). Additionally, we observed that ZDHHC11 deficiency in MEFs reduced the mRNA levels of *Ifnb1* and *Ifit1*, one IRF3-dependent gene, induced by HSV-1, which was consistent with the previous study showing ZDHHC11 modulates the innate immune response to DNA virus infection (Supplementary Figures 4J,K). Collectively, these data suggest that ZDHHC11 also played a positive role in regulating NF-κB signaling triggered by DNA virus infection.

To determinate whether ZDHHC11 is involved in NF-κB signaling *in vivo*, we first treated *Zdhhc11*^+/+^ and *Zdhhc11*^–/–^ mice with LPS and D-galactosamine by intraperitoneal injection and then measured IL-6 in their sera by ELISA. As shown in Figure 3I, the level of IL-6 protein in *Zdhhc11*^–/–^ mice was significantly lower than that in control mice. Next, we infected *Zdhhc11*^+/+^ and *Zdhhc11*^–/–^ mice with HSV-1 by intravenous injection and measured IL-6 in sera. As a result, *Zdhhc11* knockout mice also showed a significantly reduced level of IL-6 compared with control mice (Figure 3J). These results provide evidence that ZDHHC11 plays an important role in regulating NF-κB signaling *in vivo*.

### ZDHHC11 Targets TRAF6 to Regulate NF-κB Signaling

**FIGURE 4:**
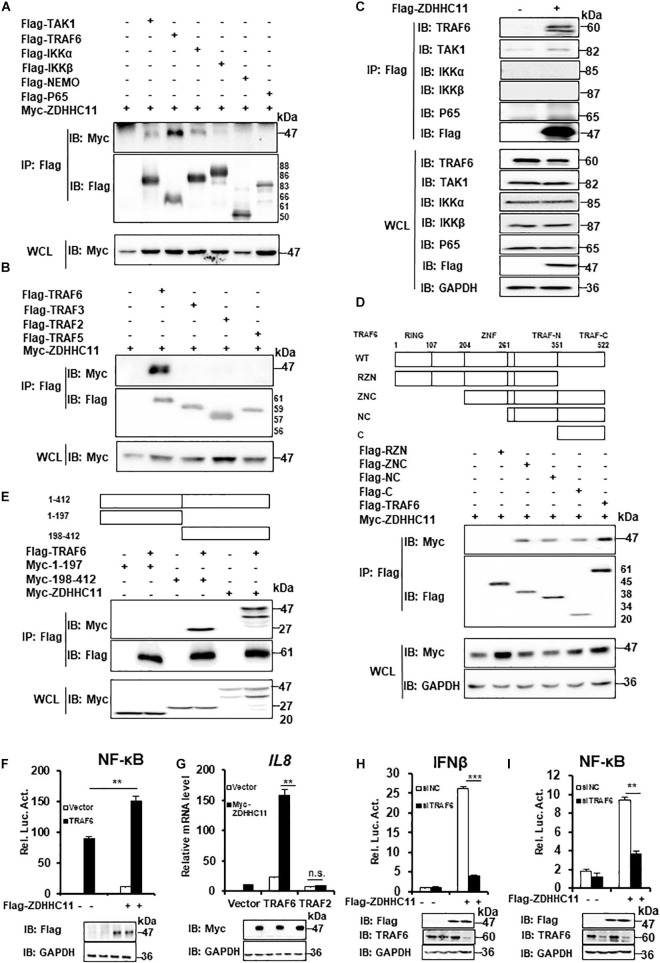
ZDHHC11 targets TRAF6 to modulate NF-κB signaling. **(A,B)** HEK293T cells were transfected with the indicated expression plasmids, After 24 h of transfection, co-immunoprecipitation were performed with anti-Flag beads and then followed by immunoblot assay with the corresponding antibodies. **(C)** HEK293T cells were transfected with an empty vector or Flag-tagged ZDHHC11 (3 μg) expression plasmid. 24 h later, co-immunoprecipitation were performed with anti-Flag beads, followed by immunoblot assay with the corresponding antibodies. **(D)** HEK293T cells were cotransfected with ZDHHC11 (2 μg) and TRAF6 (2 μg) or its truncated mutants, which is illustrated in upper panels. Co-immunoprecipitation were performed with anti-Flag beads and the results of immunoblot assay were showed in lower panels. **(E)** HEK293T cells were cotransfected with TRAF6 (2 μg) or an empty vector together with ZDHHC11 (2 μg) or its truncated mutants which is illustrated in upper panels. Co-immunoprecipitation were performed with anti-Flag beads and the results of immunoblot assay were showed in lower panels. **(F)** HEK293T cells were cotransfected with the Flag-tagged ZDHHC11 (100 ng), HA-tagged TRAF6 (20 ng) and luciferase reporter plasmid NF-κB-Luc (20 ng). 24 h later, luciferase reporter assays (upper panel) and immunoblot assays (lower panels) were performed. **(G)** HEK293T cells were cotransfected with the indicated plasmids (ZDHHC11 100 ng, TRAF6 10 ng, and TRAF2 50 ng). After 24 h, the cells were collected for qRT-PCR assays to measure the mRNA level of *IL8*. **(H,I)** HEK293T cells were transfected with a siRNA that targeted TRAF6 or a non-targeting control (NC). 24 h later, the cells were cotransfected with an empty vector or ZDHHC11 (100 ng) expression plasmid together with luciferase reporter plasmids IFNβ-Luc **(H)** or NF-κB-Luc **(I)**. After 24 h of plasmids transfection, the cells were harvested for luciferase reporter assays (upper panel) and immunoblotting (lower panels). Data shown in **(F–I)** are representative of three independent experiments (mean ± SD of duplicate experiments). **P < 0.01; ***P < 0.001 significant versus the control groups; Student’s *t*-test. All blots are representative of three independent experiments.

Our results described above demonstrated that ZDHHC11 was involved in modulating NF-κB activation stimulated by IL-1β, LPS, and DNA virus infection. Next, we tried to explore the molecular mechanism by which ZDHHC11 regulates NF-κB signaling. To identify ZDHHC11-targeted proteins, we first conducted co-immunoprecipitation (Co-IP) to test whether ZDHHC11 interacted with known components of the NF-κB pathway. We cotransfected ZDHHC11 with TAK1, TRAF6, IKKα, IKKβ, NEMO, and p65 into HEK293T cells and found that overexpressed ZDHHC11 strongly associated with TRAF6 and weakly associated with TAK1 and IKKα, whereas no interaction was detected between ZDHHC11 and IKKβ, NEMO, or p65 (Figure 4A). Because TRAF2, TRAF3, and TRAF5 have similar structures to TRAF6 and all play important roles in NF-κB signaling (Nakano et al., 1996; Park et al., 1999; Park, 2018), we next investigated whether ZDHHC11 also associated with these TRAF family members. Co-IP indicated that ZDHHC11 pull-downed TRAF6 but not TRAF2, TRAF3, or TRAF5 (Figure 4B). To further investigate the specificity of the interaction between ZDHHC11 and TRAF6, we evaluated whether Flag-tagged ZDHHC11 interacted with endogenous components of NF-κB pathway. As shown in Figure 4C, ectopic expression of ZDHHC11 was strongly associated with endogenous TRAF6, but not other components. Consistently, ZDHHC11 and TRAF6 were reciprocally co-immunoprecipitated in transfected HEK293T cells (Supplementary Figures 5A,B). These data suggest that ZDHHC11 specifically associated with TRAF6 to regulate NF-κB signaling activity.

Next, we determined which domains of ZDHHC11 and TRAF6 were responsible for their interaction. TRAF6 consists of three major domains, an N-terminal RING finger domain, Zn Finger domains, and a C-terminal TRAF domain which is further divided into a TRAF-N domain and a TRAF-C domain (Yin et al., 2009). We generated several TRAF6-truncated mutants and found that TRAF-C domain of TRAF6 was required for its interaction with ZDHHC11 (Figure 4D and Supplementary Figure 5C). ZDHHC11 mapping indicated that the C-terminal region of ZDHHC11 (198–412 aa) was involved in the association between ZDHHC11 and TRAF6 (Figure 4E). These results indicated that the association between ZDHHC11 and TRAF6 depends on a specific domain.

To further determine whether TRAF6 is a target of ZDHHC11 in NF-κB signaling, we cotransfected TRAF6 with ZDHHC11 or the empty vector together with an NF-κB-Luc reporter into HEK293T cells. The reporter assay showed that ZDHHC11 overexpression synergistically enhanced TRAF6-induced NF-κB activation (Figure 4F). Consistently, qRT-PCR showed that ZDHHC11 overexpression significantly augmented *IL8* mRNA expression induced by TRAF6, but not TRAF2 (Figure 4G). Additionally, TRAF6 knockdown by siRNA dramatically reduced the activities of IFNβ and NF-κB promoters induced by ZDHHC11 overexpression in HEK293T cells (Figures 4H,I). Taken together, these findings further suggest that TRAF6 is the target of ZDHHC11 in regulation of NF-κB signaling.

### ZDHHC11 Enhances TRAF6 E3 Activity by Augmenting TRAF6 Oligomerization

**FIGURE 5:**
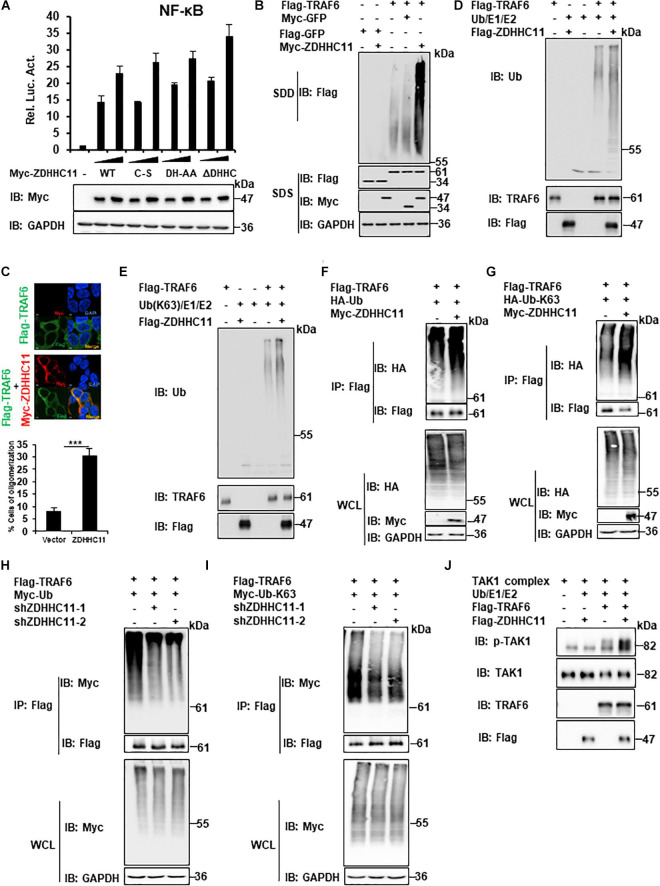
ZDHHC11 increases TRAF6 E3 activity by enhancing TRAF6 oligomerization. **(A)** HEK293T cells were cotransfected with an empty vector or expression plasmids encoding wild-type ZDHHC11 (100 ng) or its mutants (100 ng) and a NF-κB-Luc reporter plasmid (20 ng). After 24 h, reporter assays (upper panel) and immunoblot assay (lower panels) were performed. **(B)** HEK293T cells were cotransfected with the indicated expression plasmids. 24 h later, cell lysates were separated by SDD-AGE (upper panel) or SDS-PAGE (lower panels), followed by immunoblot analysis. **(C)** HEK293T cells were transfected with the indicated plasmids (500 ng/each) for 24 h, then stained with the indicated antibodies. The images were taken by confocal microscopy. Scale bars, 2 μm. The percentage of cells with larger granules was counted in 150 cells. **(D,E)** Purified TRAF6 and ZDHHC11 were incubated as indicated in a reaction mixture containing ATP, E1, E2, and Ub **(D)**, or Ub-K63 **(E)** for 1 h at 30°C, then analyzed by immunoblotting. **(F,G)** HEK293T cells were transfected with TRAF6 (1 μg) and ZDHHC11 (1 μg) or empty vector together with HA-tagged wild-type Ub (HA-Ub; **F)**, HA-Ub-K63 **(G)** plasmids (1 μg/each). At 24 h post-transfection, Co-IPs were performed with anti-Flag beads, followed by immunoblotting. **(H,I)** HEK293 C6 cells were infected with lentivirus expressing shRNA that targeted different coding regions of ZDHHC11 (shZDHHC11-1 and shZDHHC11-2) or an empty vector for 48 h. Then cells were cotransfected with TRAF6 (1 μg) and Myc-tagged wild-type Ub (Myc-Ub; **H)** or Myc-Ub-K63 **(I)** plasmids (1 μg). After 24 h of transfection, cell lysates were harvested for immunoprecipitation with anti-Flag beads, followed by immunoblotting. **(J)** Purified TRAF6, ZDHHC11, and TAK1 complex were incubated as indicated in a reaction mixture containing ATP, E1, E2, and Ub for 1 h at 30°C, then analyzed by immunoblotting. Data shown in **(A)** is representative of three independent experiments (mean ± SD of duplicate experiments). ***P < 0.001 significant versus the control groups. All blots are representative of three independent experiments.

Next, we investigated the molecular mechanisms of ZDHHC11, which regulate NF-κB by targeting TRAF6. Because ZDHHC11 is a member of the DHHC palmitoyl acyltransferase family, we examined whether palmitoyl transferase activity is required for its function in NF-κB signaling. In accordance with other members of the DHHC palmitoyl transferase family, specific aspartate-histidine (DH) and cysteine (C) residues in the DHHC domain of ZDHHC members are critical for its palmitoyl transferase activity. Therefore, we constructed several mutants of ZDHHC11, which included ZDHHC11DH/AA (D152A, H153A), ZDHHC11C/S (C155S), and ZDHHC11ΔDHHC (del 152-155 aa), in which DHHC was deleted and then examined their ability to activate NF-κB signaling. Reporter assays demonstrated that these mutants activated NF-κB signaling at similar levels as wild-type ZDHHC11 (Figure 5A). These results suggest that the palmitoyl transferase activity of ZDHHC11 was not required for its NF-κB activation.

Previous studies have demonstrated that TRAF6 oligomerization plays an important role in regulating the activity of NF-κB signaling (Ea et al., 2004; Hu et al., 2017). Therefore, we next examined whether ZDHHC11 modulates TRAF6 oligomerization. Semi-denaturing detergent agarose gel electrophoresis (SDD-AGE) assays were employed to detect TRAF6 oligomerization. As shown in Figure 5B, ZDHHC11 overexpression significantly enhanced TRAF6 oligomerization. Given that the experiments described above indicated C-terminal region of ZDHHC11 (198–412 aa) was important for the association between ZDHHC11 and TRAF6, we then examined whether C-terminal region of ZDHHC11 affects TRAF6 oligomerization. By performing SDD-AGE analysis with truncation mutants of ZDHHC11, we found that C-terminal region of ZDHHC11 (198–412 aa) could remarkably promoted TRAF6 oligomerization (Supplementary Figure 6). These results suggested that the interaction of ZDHHC11–TRAF6 was important for ZDHHC11 to enhance TRAF6 oligomerization. In addition, we conducted immunostaining and observed more and larger granules of TRAF6 when ZDHHC11 and TRAF6 were cotransfected into HEK293T cells compared with TRAF6 transfected alone (Figure 5C). These data collectively demonstrate that ZDHHC11 enhanced TRAF6 oligomerization.

Given that TRAF6 oligomerization promotes its ubiquitin ligase activity, and that ZDHHC11 enhanced TRAF6 oligomerization, we next examined whether ZDHHC11 modulated TRAF6 E3 activity using an *in vitro* ubiquitination assay. First, we used the TRAF6-Ubc13/Uev2 system with E1 and wild-type ubiquitin and found that ZDHHC11 significantly increased synthesis of ubiquitination chains (Figure 5D). Because K63-linked polyubiquitination catalyzed by TRAF6 plays an important role in the initiation of TAK1 kinase activity (Wang et al., 2001), we next examined whether ZDHHC11 regulates the synthesis of K63-linked ubiquitination chains. As shown in Figure 5E, the synthesis of K63-linked ubiquitination chains was remarkably enhanced after addition of ZDHHC11 protein to the reaction mixture. Given that TRAF6 also mediates itself polyubiquitination, next we examined whether ZDHHC11 affected the ubiquitination of TRAF6. As shown in Figures 5F–I, ZDHHC11 overexpression increased wild-type and K63-lined ubiquitination of TRAF6 which conversely were reduced by ZDHHC11 knockdown. Given that oligomerization of TRAF6 induces TAK1 activation, next we performed *in vitro* TAK1 activation assay, and found that ZDHHC11 significantly enhanced TAK1 activation (Figure 5J). Collectively, these data suggest that ZDHHC11 enhances TRAF6 E3 activity by promoting TRAF6 oligomerization and E3 ligase activity, subsequently leading to TAK1 activation.

## Discussion

The NF-κB signaling plays an essential role in inflammation and innate immunity. Additionally, increasing evidence has demonstrated that the transcription factors of NF-κB family are crucial for many steps in cancer initiation and progression (Li and Verma, 2002; Taniguchi and Karin, 2018). Here, we found that ZDHHC11, a member of the DHHC palmitoyl transferase family, positively modulated NF-κB signaling. Mechanistically, we demonstrated that ZDHHC11 enhanced TRAF6 oligomerization, which augmented its E3 ligase activation.

ZDHHC11 is a member of the DHHC palmitoyl transferase family, which has been demonstrated to play important roles in regulating STING-mediated antiviral innate immune responses (Liu et al., 2018). Consistent with this study, we also found that ZDHHC11 positively modulated anti-DNA viral innate immune responses. Interestingly, ZDHHC11 overexpression only activated the NF-κB, but not ISRE, promoter. Thus, we examined whether ZDHHC11 is involved in regulating activation of NF-κB induced by other stimuli such as IL-1β, TNFα, and LPS. qRT-PCR showed that ZDHHC11 overexpression synergistically increased the level of IL-8 mRNA induced by IL-1β, but not TNFα, in HEK293 C6 cells. ZDHHC11 knockout showed that ZDHHC11 was involved in modulating NF-κB activation induced by IL-1β, LPS, and DNA virus infection in MEFs and macrophages. Furthermore, *in vivo* experiments showed that ZDHHC11 deficiency decreased IL-6 secretion in serum induced by LPS/D-galactosamine treatment and HSV-1 infection. Notably, the palmitoyl transferase activity of ZDHHC11 was not involved in NF-κB activation. Of note, we observed that mRNA levels of ZDHHC11 was not induced by the stimulation of IL-1β, and IL-β stimulation had no effect on the association between ZDHHC11 and TRAF6 (Supplementary Figure 7). It would be interesting to address the issue of what triggers the ZDHHC11-TRAF6 binding in the future study.

Tumor necrosis factor receptor-associated factor 6 acts as an E3 ubiquitin ligase together with Ubc13-Uev1A, which catalyzes K63-linked polyubiquitination and plays a critical role in NF-κB signaling by regulating activation of the TAK1 and IKK complexes (Deng et al., 2000; Hu et al., 2017). TRAF6 oligomerization is critical for its E3 activity (Yin et al., 2009; Fu et al., 2018). In this study, Co-IP demonstrated that ZDHHC11 interacted with TRAF6 through the TRAF6-C domain that has been reported to function as a binding platform to regulate TRAF6 association with other proteins. ZDHHC11 overexpression significantly increased the level of *IL8* mRNA induced by TRAF6 overexpression, whereas knockdown of TRAF6 significantly decreased NF-κB activation induced by ZDHHC11 overexpression. These results support the notion that ZDHHC11 targets TRAF6 to regulate NF-κB signaling. Furthermore, we found that ZDHHC11 overexpression enhanced TRAF6 oligomerization, which subsequently increased E3 activity. These data suggest that ZDHHC11 positively modulates TRAF6 E3 activity by promoting its oligomerization.

Previous studies have indicated that ZDHHC11 and ZDHHC11B play critical roles in maintaining the oncogenic MYC-miR-150-MYB axis in Burkitt’s lymphoma, and ZDHHC11 may be a biomarker to identify high-risk bladder cancer patients with disease progression (Yamamoto et al., 2007; Dzikiewicz-Krawczyk et al., 2017). In our study, ZDHHC11 overexpression activated NF-κB signaling and it was involved in regulating NF-κB signaling induced by several stimuli such as Il-1β, LPS, and a DNA virus. Because NF-κB signaling plays a crucial role in cancer initiation and progression, whether the function of ZDHHC11 in cancer development is related to its regulatory role in NF-κB signaling needs to be investigated further.

In summary, we identified ZDHHC11 as a positive modulator of NF-κB signaling. ZDHHC11 interacted with TRAF6 and promoted its oligomerization, which increased E3 activity. Our data provide a new insight into understanding the regulatory mechanism of TRAF6-mediated NF-κB signaling.

## Materials and Methods

### Ethics Statement

All animal studies were performed in accordance with the recommendations in the Guide for the Care and Use of Laboratory Animals of the Ministry of Science and Technology of the People’s Republic of China. The protocols for animal studies were approved by the Committee on the Ethics of Animal Experiments of the Institute of Zoology, Chinese Academy of Sciences (Approval number: IOZ15001).

### Cell Culture and Animals

HEK293T and HeLa cells were bought from the Shanghai Cell Bank of Chinese Academy of Sciences (Shanghai, China). HEK293 C6 cells that ectopically express IL-1R were kindly provided by Dr. Zongping Xia in Zhengzhou University. Cells were cultured in Dulbecco’s modified Eagle’s medium (DMEM, Invitrogen) containing 10% (v/v) fetal bovine serum (Invitrogen) and 1% streptomycin and penicillin. Z*dhhc11*^+/+^ and *Zdhhc11*^–/–^ MEFs were isolated from 13.5-day-old embryos of Z*dhhc11*^+/+^ and *Zdhhc11*^–/–^ mice (The Jackson Laboratory). MEFs were cultured in complete DMEM containing 1 mM sodium pyruvate, 10 μM L-glutamine, 10 μM β-mercaptoethanol, and 1% non-essential amino acids. BMDMs were prepared as described previously (Li et al., 2013). Genomic DNA was extracted from 2-week-old mouse tails or cells for genotyping, followed by PCR analyses in accordance with the instructions from The Jackson Laboratory. The sequence of primers for genotyping is as follows:

#1 (5′–3) CTGCCATACACCTAAATGCCTCAGC;
#2 (5′–3) TTTCGGAGCTGAAAAGCCAAGAAGG;
#3 (5′–3) ACTTGCTTTAAAAAACCTCCCACA;
#4 (5′–3) CCACATACCACACAGACATACACAGC.

### Plasmids

Flag-tagged ZDHHC11, TRAF2, TRAF3, TRAF5, TRAF6, TAK1, IKKα, IKKβ, NEMO, P65 and; Myc-tagged ZDHHC11 were cloned into pcDNA3 or pEF vector. ZDHHC11 and TRAF6 mutants were generated by PCR using Pfu DNA polymerase. IFN-β, NF-κB, and ISRE luciferase reporter plasmids have been described previously (Zhao et al., 2012).

### Antibodies

Rabbit anti-Flag was purchased from Sigma. Rabbit and mouse anti-Myc antibodies were purchased from MBL. Rabbit anti-p-P65 (Ser536, 3033), anti-TAK1 (5206), anti-p-TAK1 (T184/187, 4508), anti-IKKα (61294), anti-IKKβ (8943), anti-p-IKKα/β (S176/180, 2697), and mouse anti-p-IκBα (Ser32/36, 9246) antibodies were from Cell Signaling Technology. Mouse anti-p65 (SC-8008), anti-TRAF2 (SC-876), anti-ubiquitin (SC-8017), and rabbit anti-IκBα (SC-371) were from Santa Cruz Biotechnology. Rabbit anti-TRAF6 (ab40675) was from Abcam. Mouse anti-GAPDH (KM9002) and anti-Tubulin (KM9007) were from Sungene Biotechnology.

### Transfection and Luciferase Assay Reporter

HEK293T cells were cotransfected with the indicated expression plasmids or an empty vector with a Renilla reporter plasmid and luciferase reporter plasmid that encoded IFNβ-Luc, NF-κB-Luc, or ISRE-Luc. The empty control plasmid was added to ensure that each transfection obtains the same amount of total DNA. 24 h after transfection, the cells were lysed for luciferase activity, and transfection efficiency was normalized to Renilla activity (Tao et al., 2020).

### Co-IP and Immunoblot Assay

The Co-IP methods have been described previously (Zhao et al., 2012). Briefly, cells were lysed in lysis buffer with protease inhibitor cocktail (Roche) and incubated at 4°C with anti-Flag agarose beads (Sigma) or anti-Myc magnetic beads (Bimake) for 4 h. The complexes were washed 3–4 times and subjected to immunoblot assay. For detecting multiple phosphoproteins, the cells were directly lysed in 1x SDS-PAGE sample loading buffer (50 mM Tris pH 6.8, 1% mercaptoethanol, 2% SDS, 0.01% bromophenol blue, and 10% glycerol). Immunoblotting was conducted using standard procedures.

### SDD-AGE Assay

Cells were transfected as indicated and then lysed in lysis buffer (1% NP40, 50 mM Tris–HCl, 150 mM NaCl, 1 mM EDTA, and 10% glycerol) with protease inhibitor cocktail (Roche) for 30 min at 4°C. The cell lysates were centrifugated at 10,000 rpm for 10 min. The SDD-AGE assay was performed as described previously (Hou et al., 2011). Briefly, the supernatants were resuspended in 1x sample buffer (0.5x TBE, 2% SDS, 10% glycerol, and 0.01% bromophenol blue), loaded on 1.5% agarose gel and electrophoresis was performed in the running buffer (1x TBE and 0.1% SDS) with a constant voltage of 110 V for 45 min at 4°C, followed by immunoblotting.

### Immunofluorescence

HEK293T cells were cultured in gelatin-coated 12-well plates overnight, and then transfected with the indicated plasmids. After 24 h, the cells were washed with phosphate-buffered saline, fixed with 4% paraformaldehyde for 10 min, permeabilized with 0.2% Triton X-100 for 15 min, and then blocked with 5% (w/v) bovine serum albumin for 30 min, followed by incubating with primary and secondary antibodies. Imaging was performed under a Zeiss LSM 710 META laser scanning confocal system and ANDOR CR-DFLY-505 confocal microscope equipped with a sCMOS Zyla 4.2 plus camera.

### *In vitro* Ubiquitination Assays

HEK293T cells were transfected with Flag-ZDHHC11 or Flag-TRAF6 and then cultured for 36 h. The cells were lysed with lysis buffer (0.5% NP40, 20 mM Tris–HCl pH7.5, 150 mM NaCl, 10% glycerol, and 1 mM EDTA) with protease inhibitor cocktail (Roche) and then purified with anti-Flag beads. The proteins were eluted by a Flag peptide after extensive washing with buffer (0.5% NP40, 20 mM Tris–HCl pH 7.5, 500 mM NaCl, 10% glycerol, and 1 mM EDTA). *In vitro* ubiquitination assays were performed in a reaction mixture containing recombinant E1, Ubc13/Uev2, TRAF6, and ubiquitin (WT or K63-linked) in ATP buffer in the presence or absence ZDHHC11 protein. The reaction was incubated at 30°C for 1 h and terminated by addition of denaturing sample buffer, followed by 95°C heating for 5 min. The samples were resolved on 6–18% or 10% SDS-PAGE gels, followed by immunoblotting with indicated antibodies.

### *In vitro* TAK1 Kinase Activation Assays

HEK293T cells were infected with lentivirus expressing PCDH-Flag-TAK1. At 100 h post-infection, the cells were lysed with lysis buffer (0.5% Tritonx-100, 20 mM Tris–HCl pH7.5, 150 mM NaCl, 10% glycerol, and 1 mM EDTA) with protease inhibitor cocktail (Roche) and then purified with anti-Flag beads. The proteins were eluted by a Flag peptide after washing with buffer (0.5% Tritonx-100, 20 mM Tris–HCl pH 7.5, 150 mM NaCl, 10% glycerol, and 1 mM EDTA). TRAF6 and ZDHHC11 protein were purified as described in ubiquitination assays. *In vitro* TAK1 kinase activation assays were performed in a reaction mixture containing recombinant E1, Ubc13/Uev2, TRAF6, TAK1 complex and ubiquitin in ATP buffer with or without ZDHHC11 protein. The reaction was incubated at 30°C for 1 h and terminated by addition of denaturing sample buffer, followed by 95°C heating for 5 min. The samples were resolved on 10% SDS-PAGE gels and then analyzed by immunoblotting with the indicated antibodies.

### Target Gene Knockdown by RNA Interference and Lentivirus-Mediated shRNA

HEK293T cells were transfected with siRNA that targeted TRAF6 or a non-targeting control (NC) at a final concentration of 30 nM by the standard calcium phosphate transfection method. 24 h after transfection, the cells were transfected with the indicated plasmids using Lipofectamine 3000 (Invitrogen) for 24 h and then harvested for luciferase reporter assays and immunoblot analysis. The sequence for human TRAF6 siRNA were as follows (5′–3′): CUGUGCUGCAUCAAUGGCA.

HEK293 C6 and HeLa cells were infected with lentivirus that targeted two different regions of human *ZDHHC11* (shZDHHC11-1 and shZDHHC11-2) or an empty vector for 48 h. The cells were untreated or treated with IL-1β for the indicated times, followed by subsequent experiments. Knockdown efficiency was determined by qRT-PCR. The shRNA sequences against human *ZDHHC11* were as follows (5′–3′): shZDHHC11-1: CTCCAATGTCAGACTCATGAA; shZDHHC11-2: CCACCTTTGAGTAT CTCATTA.

### qRT-PCR

Total RNA was isolated from cells with TRIZOL reagent (Invitrogen), cDNA was synthesized with a SuperScript III First-Strand cDNA Synthesis kit (Invitrogen). qRT-PCR was performed using SYBR Green Master Mix (Thermo Fisher) and Bio-Rad CFX connect system. Data were normalized to the abundance of GAPDH mRNA, and shown with the relative abundance of mRNA compared with the control group. The primers used were listed as follows (5′–3′):

h*ZDHHC11*-S GGTGCAGACCCTGATAGTCG
h*ZDHHC11*-AS GCACGTATGGATCTTTCCTCAC
h*IL8*-S ATAAAGACATACTCCAAACCTTTCCAC
h*IL8*-AS AAGCTTTACAATAATTTCTGTGTTGGC
h*TNF*α-S CTGCCCCAATCCCTTTATT
h*TNF*α-AS CCCAATTCTCTTTTTGAGCC
h*IFIT1*-S TACCTGGACAAGGTGGAGAA
h*IFIT1*-AS GTGAGGACATGTTGGCTAGA
h*GAPDH*-S ATGACATCAAGAAGGTGGTG
h*GAPDH*-AS CATACCAGGAAATGAGCTTG
m*Il6*-S TCGGAGGCTTAATTACACATGTTCT
m*Il6*-AS TGCCATTGCACAACTCTTTTCT
m*Tnf*α-S TCCCCAAAGGGATGAGAAGTT
m*Tnf*α-AS GTTTGCTACGACGTGGGCTAC
m*Gapdh*-S AACTTTGGCATTGTGGAAGG
m*Gapdh*-AS ACACATTGGGGGTAGGAACA
m*Ifnb1*-S ATGGTGGTCCGAGCAGAGAT
m*Ifnb1*-AS CCACCACTCATTCTGAGGCA
m*Il-1*β-S AAAGCCTCGTGCTGTCGGACC
m*Il-1*β*-*AS CAGGGTGGGTGTGCCGTCTT.

### *In vivo* LPS Treatment and Viral Infection and ELISA

*Zdhhc11*^+/+^ and *Zdhhc11*^–/–^ mice were treated with LPS (3 μg/kg) and D-galactosamine (250 mg/kg) via intraperitoneal injection. Sera were collected at 1 and 3 h after injection to measure IL-6 by a mouse IL-6 ELISA kit (BD Biosciences) following with the manufacturer’s instructions. *Zdhhc11*^+/+^ and *Zdhhc11*^–/–^ mice were infected with HSV-1 via tail vein injection at 2 × 10^7^ plaque-forming units (PFU)/mouse. Sera were collected at 4 and 8 h after infection to measure IL-6 production by an ELISA kit.

### Statistical Analysis

The data are presented as means ± SD and two-tailed Student’s *t*-test was used to examine significant differences between values under different experimental conditions. For all tests, *p* values < 0.05 were considered statistically significant.

## Data Availability Statement

The raw data supporting the conclusions of this article will be made available by the authors, without undue reservation.

## Ethics Statement

The animal study was reviewed and approved by the Institute of Zoology, Chinese Academy of Sciences.

## Author Contributions

EL, DC, and QS designed the experiments and wrote the manuscript. EL, JS, JY, LL, QY, JZe, and JZh performed the experiments. EL and QS performed data analysis. All authors contributed to the article and approved the submitted version.

## Conflict of Interest

The authors declare that the research was conducted in the absence of any commercial or financial relationships that could be construed as a potential conflict of interest.

## Publisher’s Note

All claims expressed in this article are solely those of the authors and do not necessarily represent those of their affiliated organizations, or those of the publisher, the editors and the reviewers. Any product that may be evaluated in this article, or claim that may be made by its manufacturer, is not guaranteed or endorsed by the publisher.

## Supplementary Material

Supplementary Figure 1 Identification of ZDHHC11 as a positive regulator in NF-κB signaling.

Supplementary Figure 2 ZDHHC11 overexpression activates NF-κB downstream genes in HeLa cells.

Supplementary Figure 3 ZDHHC11 knockdown reduces NF-κB activation stimulated by IL-1β in HeLa cells.

Supplementary Figure 4 *Zdhhc11* deficiency reduces NF-κB activation stimulated by IL-1β, LPS, and DNA virus infection.

Supplementary Figure 5 TRAF6 interacts with ZDHHC11 through its TRAF C domain.

Supplementary Figure 6 C-terminal (198-412aa) region of ZDHHC11 plays an important role in enhancing TRAF6 oligomerization.

Supplementary Figure 7 IL-1β treatment has no effect on the mRNA level of ZDHHC11 and association with TRAF6.
