# Corrigendum: ZDHHC11 Positively Regulates NF-κB Activation by Enhancing TRAF6 Oligomerization

**DOI:** 10.3389/fcell.2021.761639

**Published:** 2021-09-16

**Authors:** Enping Liu, Jiawei Sun, Jing Yang, Lin Li, Qili Yang, Jiuqin Zeng, Jiayu Zhang, Dahua Chen, Qinmiao Sun

**Affiliations:** ^1^State Key Laboratory of Membrane Biology, Institute of Zoology, Chinese Academy of Sciences, Beijing, China; ^2^Institute of Stem Cells and Regeneration, Chinese Academy of Sciences, Beijing, China; ^3^School of Life Sciences, University of Chinese Academy of Sciences, Beijing, China; ^4^Institute of Physical Science and Information Technology, Anhui University, Hefei, China; ^5^Institute of Biomedical Research, Yunnan University, Kunming, China

**Keywords:** ZDHHC11, NF-κB, TRAF6, oligomerization, inflammation

During the production process, the supplementary data files which include the raw data and **Figure 4** were incorrectly uploaded. The correct supplementary data files have been uploaded.

The authors apologize for this error and state that this does not change the scientific conclusions of the article in any way. The original article has been updated.

## Publisher's Note

All claims expressed in this article are solely those of the authors and do not necessarily represent those of their affiliated organizations, or those of the publisher, the editors and the reviewers. Any product that may be evaluated in this article, or claim that may be made by its manufacturer, is not guaranteed or endorsed by the publisher.

