# Supplementary figures and images for: ZDHHC11 Positively Regulates NF-κB Activation by Enhancing TRAF6 Oligomerization
Source: Front Cell Dev Biol. 2021 Aug 19;9:710967. doi: 10.3389/fcell.2021.710967 (PMC8417235; doi:10.3389/fcell.2021.710967)

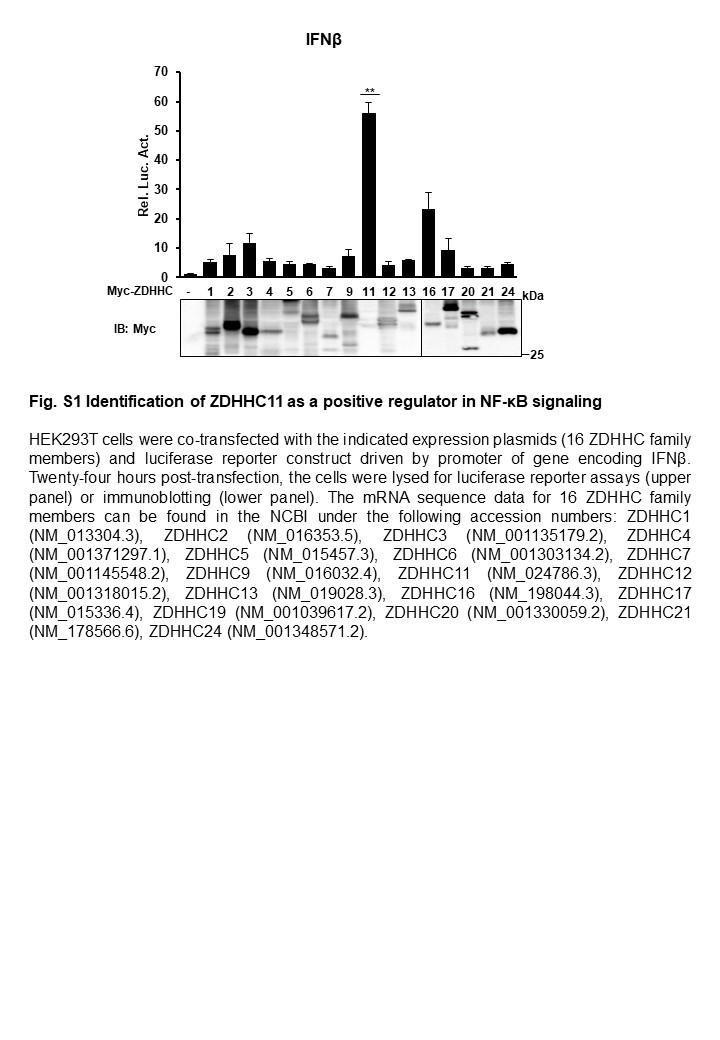

Supplement: Supplementary Figure 1 — Identification of ZDHHC11 as a positive regulator in NF-κB signaling. [file Image_1.jpeg]

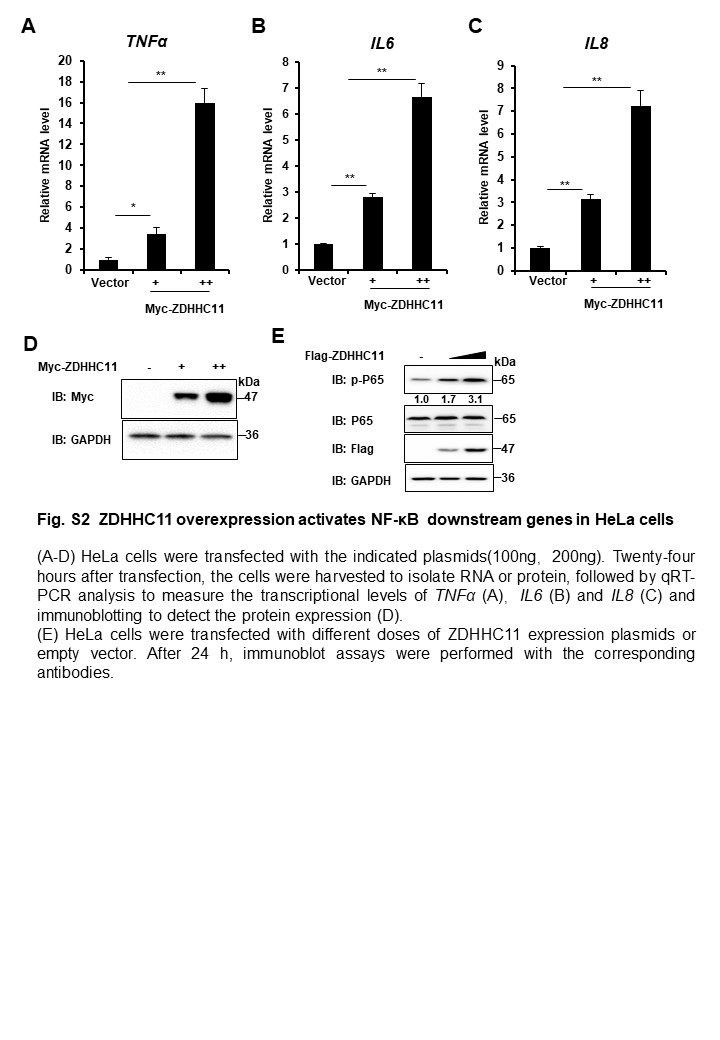

Supplement: Supplementary Figure 2 — ZDHHC11 overexpression activates NF-κB downstream genes in HeLa cells. [file Image_2.jpeg]

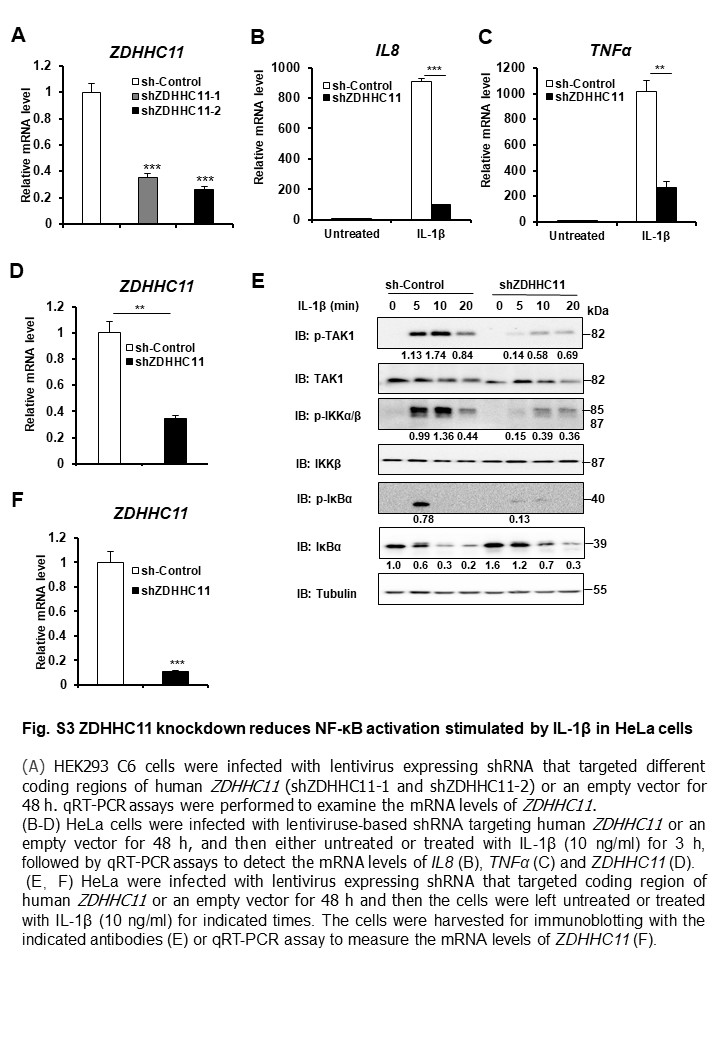

Supplement: Supplementary Figure 3 — ZDHHC11 knockdown reduces NF-κB activation stimulated by IL-1β in HeLa cells. [file Image_3.jpeg]

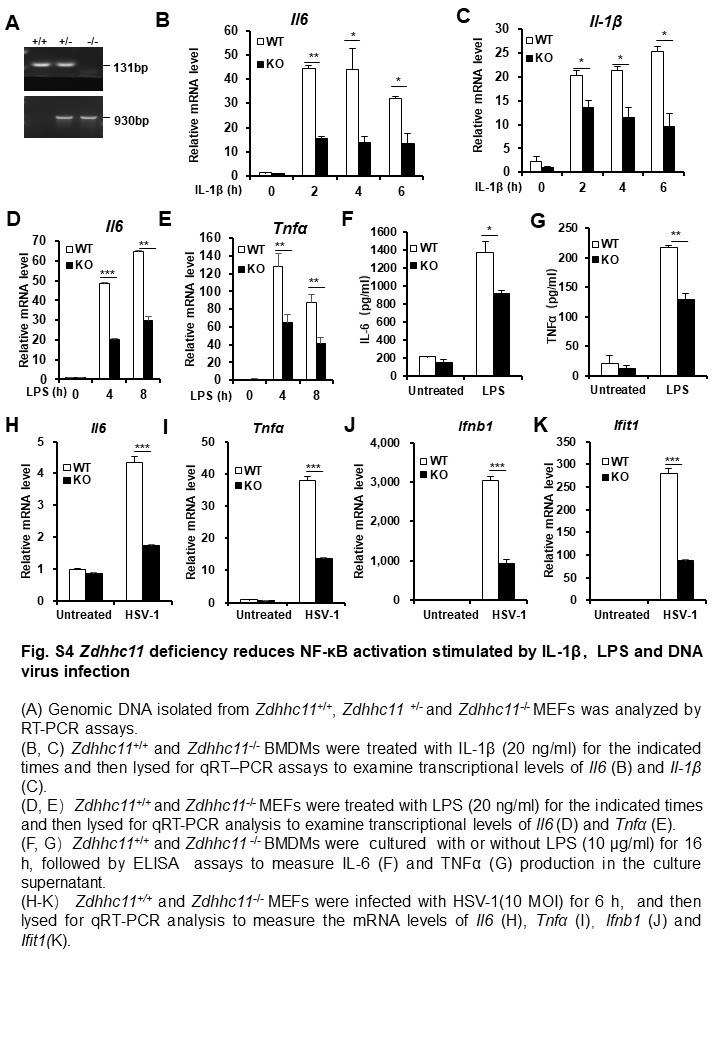

Supplement: Supplementary Figure 4 — Zdhhc11 deficiency reduces NF-κB activation stimulated by IL-1β, LPS, and DNA virus infection. [file Image_4.jpeg]

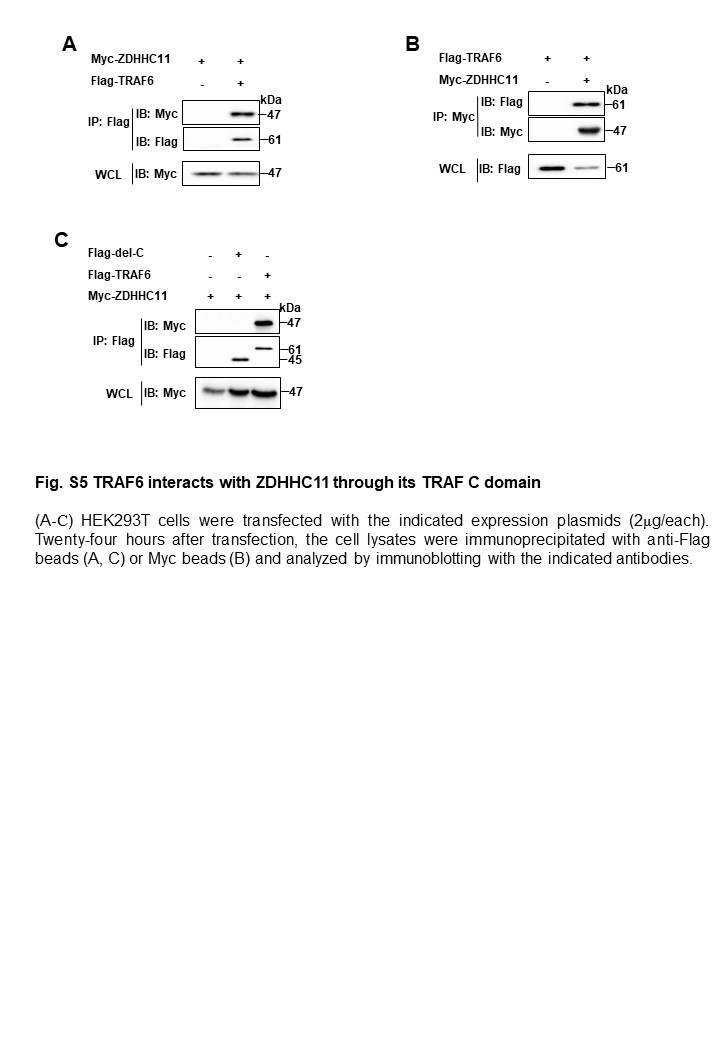

Supplement: Supplementary Figure 5 — TRAF6 interacts with ZDHHC11 through its TRAF C domain. [file Image_5.jpeg]

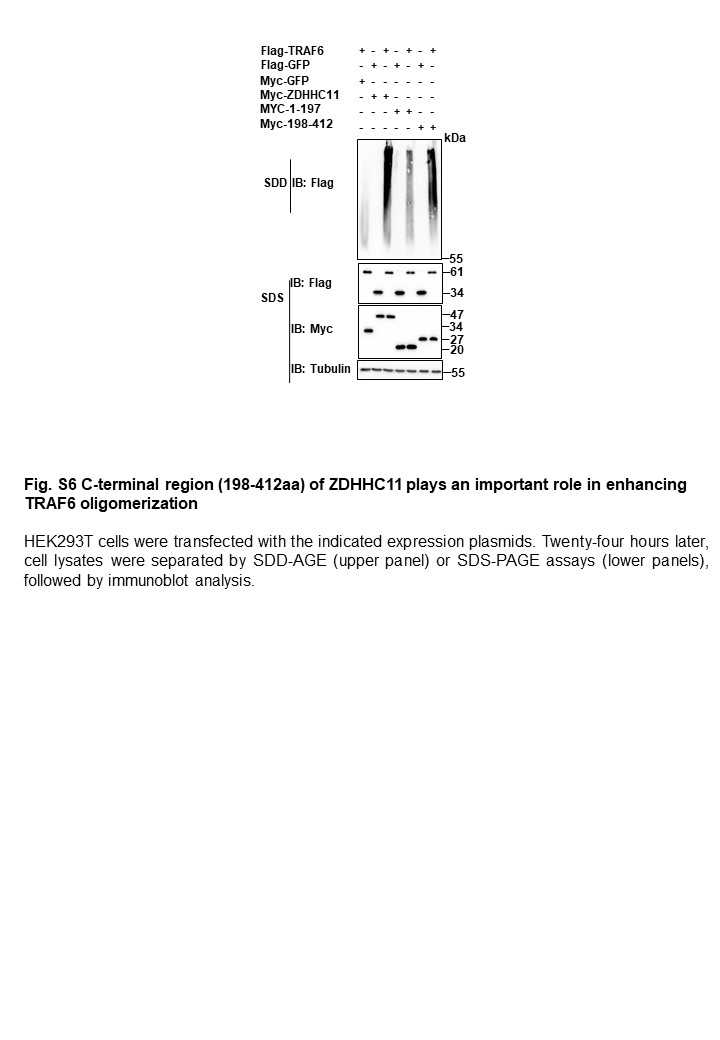

Supplement: Supplementary Figure 6 — C-terminal (198-412aa) region of ZDHHC11 plays an important role in enhancing TRAF6 oligomerization. [file Image_6.jpeg]

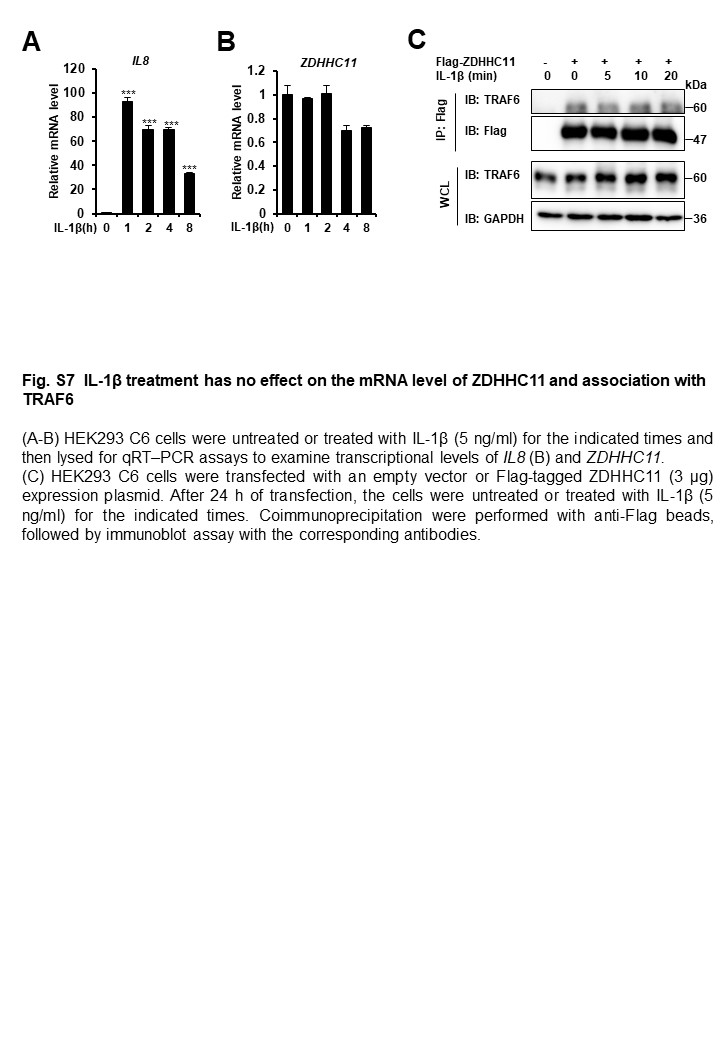

Supplement: Supplementary Figure 7 — IL-1β treatment has no effect on the mRNA level of ZDHHC11 and association with TRAF6. [file Image_7.jpeg]
